# Trace-LogVector-Based Relational Retrieval for Conversational System Log Analysis

**DOI:** 10.3390/s26061806

**Published:** 2026-03-12

**Authors:** Sun-Chul Park, Young-Han Kim

**Affiliations:** 1 Department of Artificial Intelligence IT Convergence, Soongsil University, Seoul 06978, Republic of Korea; sunpark@soongsil.ac.kr; 2 School of Electronic Engineering, Soongsil University, Seoul 06978, Republic of Korea

**Keywords:** system log analysis, retrieval-augmented generation, relational log representation, Trace-LogVector, information retrieval, IoT cloud systems, sensor-driven systems

## Abstract

**Highlights:**

**What are the main findings?**

- A relational log representation, Trace-LogVector (TLV), consistently improves retrieval accuracy in RAG-based system log analysis compared to single-chunk representations.
- CARD-based multi-chunk construction preserves execution contexts and entity relationships, leading to substantial gains in Hit@5 and MRR@5 metrics.

**What are the implications of the main findings?**

- Retrieval performance in conversational system log analysis is strongly influenced by the granularity and structure of log representations, not solely by the embedding model or retrieval algorithm.
- Relational log representations provide an effective design principle for applying RAG to sensor-driven and cloud-based system analysis tasks.

**Abstract:**

System logs generated in IoT-based and sensor-driven cloud environments encode execution traces and complex relationships among services, functions, and data stores. In many IoT deployments, telemetry is pre-processed at the edge and then integrated into backend services (e.g., application servers and databases) for analytics and operations. During this integration, service executions record relational dependencies (e.g., function-to-data-store interactions) as operational logs (or aggregated statistics), which constitute key evidence for operating sensor-driven services. We therefore evaluate TLV using publicly reproducible backend execution logs as a representative backend model and discuss the generality and limitations of this choice. However, most existing retrieval-augmented generation (RAG) approaches remain document-centric, representing logs as flat textual chunks that fail to preserve execution flow and entity relationships, which are critical for diagnosing complex service execution pipelines in sensor-driven cloud backends. In this study, we propose Trace-LogVector (TLV), a relational log representation that transforms system logs into trace-level retrieval units while explicitly preserving execution order and entity interactions. TLV is constructed based on the Chunk as Relational Data (CARD) design principle, which represents execution flows using entity-centric multi-chunk structures rather than single aggregated text chunks. To evaluate the impact of relational log representation, we conduct controlled experiments comparing single-chunk and CARD-based multi-chunk TLV under identical embedding and retrieval settings. Retrieval performance is quantitatively assessed using Hit@5 and Mean Reciprocal Rank at 5 (MRR@5). Experimental results show that the proposed multi-chunk TLV achieves a Hit@5 of 1.000 and an MRR@5 of 0.900, consistently outperforming the single-chunk baseline across all evaluation queries. These findings demonstrate that preserving execution contexts and entity relationships as relational retrieval units is a key factor in improving RAG-based system log analysis for monitoring and diagnosing large-scale sensor networks and cloud systems.

## 1. Introduction

Recent advances in Large Language Models (LLMs) have led to substantial improvements in natural language understanding and generation, enabling their successful adoption across a wide range of industrial and societal applications [1,2]. In particular, Retrieval-Augmented Generation (RAG) architectures, which augment LLMs with external knowledge sources, have proven effective in improving response accuracy and mitigating hallucinations, especially when integrated with vector databases for domain-specific knowledge retrieval [3]. Consequently, LLM-based RAG systems have been widely adopted for tasks such as document authoring, customer support, and enterprise knowledge assistance [4].

However, most existing RAG approaches are primarily tailored for document-centric and unstructured text data. Their applicability to system analysis and operational domains remains limited, despite the growing demand for intelligent automation in these areas. In IoT-based and sensor-driven cloud environments, massive volumes of logs, events, and sensor data are continuously generated, reflecting complex system execution and state transitions. Nevertheless, operational analysis in such environments still relies heavily on traditional and manual approaches.

In practice, critical analysis tasks—such as identifying root causes of faults, tracing execution flows, and understanding service dependencies—are typically performed through Command-Line Interfaces (CLIs), Structured Query Language (SQL) queries, or manual inspection by domain experts [5]. Although these methods are effective for experienced operators, they impose high technical entry barriers and limit the reusability and scalability of analysis results. As a result, system log analysis in large-scale IoT cloud environments remains a challenging domain for non-expert users [6].

A fundamental reason for this limitation lies in the structural mismatch between system logs and document-centric RAG designs. Unlike independent textual records, system logs represent cumulative execution traces (hereinafter referred to as execution flows) involving interactions among multiple entities, such as sensors, services, and databases. Conventional flat text chunking fails to preserve execution order and inter-entity relationships, which are essential for answering analysis-oriented queries that require tracing causal paths and operational dependencies.

To address the need for interactive system analysis, recent studies have explored architectural extensions such as the Model Context Protocol (MCP), which enables LLMs to interact with external tools and system resources in real time [7]. While such approaches demonstrate promise for system querying and automated execution, they remain limited in their ability to interpret and analyze execution flows and entity relationships embedded within logs. This observation suggests that conversational system analysis requires not only real-time execution capabilities but also retrieval mechanisms grounded in relational log representations.

Motivated by this insight, this study focuses on the design and evaluation of a relational RAG approach for system log analysis in IoT and sensor-driven cloud environments. We propose Trace-LogVector (TLV), a trace-level log representation that preserves execution flows and entity relationships, and adopt the Chunk as Relational Data (CARD) principle to construct entity-centric multi-chunk retrieval units. By quantitatively comparing single-chunk and multi-chunk retrieval strategies under identical embedding and retrieval conditions, this work investigates how relational log representation influences retrieval accuracy and precision in system log analysis.

**Figure 1:**
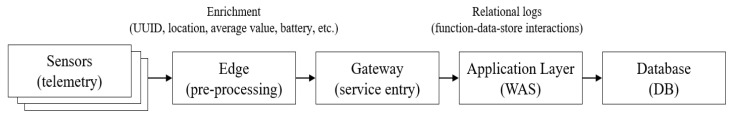
IoT telemetry integration pipeline (edge → gateway → backend services) and the resulting relational execution logs used for operational analysis.

Although this study is motivated by IoT/sensor-driven environments, the operational questions we target—such as tracing execution paths and identifying data-store interactions—are typically answered from the backend side, where telemetry is incorporated into service-level processing. In other words, we target backend relational execution logs generated as IoT telemetry is integrated into application services, rather than the raw sensor readings alone. To ensure reproducibility and to capture high concurrency and asynchronous event interleaving—conditions that amplify context pollution in naive chunking—we use a publicly reproducible backend execution-log dataset as a representative backend model. We do not claim that this dataset is identical to device-side IoT logs; instead, it serves as a controlled proxy for backend log characteristics that also appear in modern IoT telemetry pipelines. As illustrated in Figure 1, we focus on the backend integration layer where telemetry is transformed into service executions and relational logs.

## 2. Materials and Methods

### 2.1. System Log Parsing

System logs constitute a primary source of data for understanding system execution behavior and internal states, and they play a critical role in fault diagnosis, performance analysis, and operational monitoring. Traditionally, system log analysis has focused on preprocessing techniques that transform unstructured textual logs into structured representations to enable large-scale analysis. Representative approaches include automated log parsing methods such as DRAIN, which employs a fixed-depth parse tree to group log messages into templates, thereby improving the efficiency and scalability of log processing [8].

While such log parsing techniques are effective for extracting log patterns and supporting tasks such as anomaly detection, they exhibit inherent limitations in representing relationships among entities embedded within log messages. In particular, conventional parsing approaches treat logs as independent event-level records, without explicitly modeling invocation relationships or execution flows across system components. As a result, analyses based solely on parsed log templates remain insufficient for understanding interactions among system entities and do not directly support higher-level analysis tasks, such as relational retrieval or query-driven reasoning over execution traces.

### 2.2. Log Embedding

In the field of system log analysis, early studies primarily focused on modeling logs using quantitative features or temporal patterns for anomaly detection. Representative approaches such as LogAnomaly perform unsupervised anomaly detection by learning sequential patterns from log event sequences combined with statistical characteristics [9]. While effective for identifying abnormal system behavior, these methods emphasize pattern learning for anomaly detection rather than representing logs as semantic or relational units in a vector space.

More recently, research efforts have explored embedding-based representations of logs by adapting techniques from natural language processing and graph representation learning. For example, Log2Vec models relationships among log events, templates, and parameters as a heterogeneous graph and applies graph embedding techniques to capture structural correlations among logs in a vector space, enabling downstream tasks such as classification and clustering for security threat analysis [10].

Despite these advances, most existing log embedding approaches treat logs as single, flattened representation units and do not explicitly preserve structural relationships or entity-level interactions across execution traces. In many cases, inter-entity dependencies are abstracted at the same embedding level or implicitly encoded as contextual information, which limits the expressiveness of embeddings for analysis tasks that require relational reasoning. Consequently, these limitations hinder the direct application of conventional log embeddings to relation-aware retrieval and query-driven analysis scenarios.

### 2.3. LLM-Based Retrieval-Augmented Generation

Since the emergence of large language models (LLMs), retrieval-augmented generation (RAG) architectures have been extensively studied as a means of improving the accuracy and reliability of generated responses by grounding LLM outputs in external knowledge sources. In a typical RAG pipeline, a pre-constructed document collection or knowledge base is embedded into a vector space, and relevant information is retrieved in response to a user query and subsequently used to guide response generation. This paradigm has demonstrated strong effectiveness in domain-specific question answering and knowledge management tasks [11].

However, most existing RAG studies primarily target document-centric and unstructured textual knowledge. Their applicability to system logs, which consist of multiple interacting entities and execution relationships, remains relatively limited. Unlike static documents, system logs encode execution traces that reflect dynamic interactions among system components, making them poorly suited to flat document-style retrieval units.

Furthermore, conventional RAG architectures assume that the retrieval corpus is constructed in advance and remains relatively static during inference. This assumption introduces structural constraints when applying RAG to system analysis scenarios that require reflection of evolving system states or immediate execution-driven analysis. As a result, directly applying existing RAG designs to system log analysis is insufficient for supporting relation-aware retrieval and analysis-oriented queries in sensor-driven cloud environments.

### 2.4. Evaluation of RAG Performance

We formulate the task as an information retrieval (IR) problem for LLM-assisted system analysis: given a natural-language query, retrieve the most relevant execution unit(s) and entity relations as evidence. This differs from anomaly detection, forecasting, or classification settings that assume a predefined label space; our queries are open-ended and require retrieving supporting relational context rather than predicting a fixed class.

Research on evaluating the performance of LLM-based retrieval-augmented generation (RAG) systems has expanded rapidly in recent years, with evaluation perspectives extending beyond linguistic quality toward the integration of information retrieval performance. Early studies primarily relied on n-gram-based metrics such as BLEU and ROUGE, as well as F1 scores, to assess the surface-level similarity between generated responses and reference texts. However, these metrics do not directly reflect whether relevant documents were successfully retrieved or how effectively relevant information was ranked among multiple candidates, particularly in retrieval-intensive scenarios [12].

To address these limitations, recent RAG evaluation studies have increasingly adopted metrics from the information retrieval (IR) domain. Metrics such as Hit@K and Mean Reciprocal Rank (MRR) quantitatively measure whether the correct answer is included within the Top-K retrieved candidates and how highly it is ranked, respectively, enabling a more direct assessment of retrieval-stage performance [13,14]. Given the central role of retrieval modules in RAG pipelines, IR-based evaluation provides a structurally more appropriate framework than generation-focused metrics alone.

This distinction is particularly important in system log analysis domains, where analysis targets often correspond to explicit entities or relationships rather than free-form textual responses. In such contexts, the reliability of analysis depends more critically on whether the correct information is retrieved than on the linguistic similarity of generated outputs. Nevertheless, many existing RAG studies, which primarily focus on document-based question answering, do not provide sufficient quantitative comparisons of retrieval performance for relational data such as system logs.

In this study, we evaluate the performance of the proposed TLV-based RAG using Hit@K and MRR metrics. By quantitatively comparing single-chunk and multi-chunk retrieval strategies, we analyze how chunk construction that preserves entity relationships influences recall and ranking precision at the retrieval stage. This evaluation perspective emphasizes the role of accurate information retrieval as a primary determinant of analytical reliability in system log analysis, thereby providing a more direct and domain-appropriate assessment of RAG performance.

### 2.5. Trace-Based Log Analysis and Execution Flow Modeling

Business Process Management (BPM) event logs typically represent cases and activities for process discovery and bottleneck analysis. In contrast, TLV/CARD represent trace-level execution and explicit entity interactions (e.g., function–data-store relations) as retrieval units, with the primary goal of semantic evidence retrieval for answering operational questions in a RAG pipeline.

In IoT-based and sensor-driven cloud environments, large numbers of sensor devices and microservices interact continuously, generating massive volumes of event logs and state logs. Existing studies have primarily focused on anomaly detection or time-series analysis of sensor data, while system log analysis has often remained at the level of event-wise statistical methods or rule-based detection. Such approaches are limited in their ability to interpret execution flows formed by the interplay of sensor events and service invocations from a relational perspective or to support query-driven exploration of system behavior. Motivated by these limitations, this study extends prior work by adopting a relational and retrieval-oriented perspective on execution-flow-level log analysis in IoT cloud environments.

Recent advances in system log analysis have increasingly moved beyond individual log events toward reconstructing execution flows and relationships among system components embedded within logs. These approaches aim to improve causal understanding of system behavior by defining execution paths—such as request-level or transaction-level traces—as primary analysis units rather than isolated log messages.

In distributed systems, distributed tracing techniques have been widely adopted to track service invocation paths based on request identifiers (trace IDs), enabling the analysis of inter-service call relationships and latency characteristics. Systems such as Jaeger and Zipkin provide effective tools for visualizing service call flows and identifying performance bottlenecks in microservice architectures [15,16]. In addition, several studies have modeled structural relationships among remote procedure calls (RPCs), service boundaries, and database accesses using call graphs or dependency graphs, capturing execution dependencies across system components [17].

These trace-based approaches distinguish themselves from traditional log parsing or embedding methods by interpreting logs as structured data that encode execution flows and relational information rather than as collections of independent textual events. However, existing trace-based log analysis research has primarily focused on performance bottleneck analysis, fault localization, or visualization-oriented tool design. Their use of traces has largely been confined to diagnostic outputs, and relatively limited attention has been paid to leveraging traces as direct representation units for conversational query answering or retrieval-based analysis.

The Trace-LogVector (TLV) proposed in this study extends existing trace-based analysis paradigms by introducing a relational log representation that transforms execution flows into retrieval units for RAG-based system analysis. Rather than treating traces solely as analytical artifacts or visualization targets, TLV converts execution flows into vector representations that explicitly preserve entity relationships and execution order. By enabling traces to function as searchable and retrievable units, TLV reframes system log analysis as an interactive information retrieval problem, thereby differentiating this work from prior trace-centric studies.

### 2.6. Trace-LogVector Representation

In system log analysis, trace logs can be interpreted not as isolated records of individual events but as sequential records of execution activities generated according to internal processing flows triggered by service invocations. However, most existing log embedding approaches fail to adequately capture the continuity of execution flows and the relationships among log events, as they predominantly vectorize individual log messages as independent textual units. While such approaches can measure semantic similarity between logs, they are limited in supporting system analysis tasks that require exploration based on invocation relationships or data flow dependencies.

To address these limitations, this study introduces Trace-LogVector (TLV), a relational log representation that explicitly incorporates entity relationships and execution flows embedded within system logs. TLV extends conventional log embedding methods by constructing a single relational representation unit from multiple log messages belonging to the same execution trace, rather than embedding each log message independently. In this study, a trace is defined not as a simple aggregation of raw log messages but as a transaction-level execution flow derived through service call analysis techniques proposed in prior work [18]. These call analysis methods are designed to structurally extract service invocation relationships, function calls, and database access patterns, forming the basis for trace-level execution modeling.

Based on this definition, TLV transforms trace-level execution flows into structured representations that preserve relational information and maps them into a vector space. By doing so, system logs can be utilized as execution-centric analysis units rather than as collections of unstructured text. This representation is particularly well suited for RAG-based retrieval, as many system analysis queries target execution paths or operational behaviors rather than individual log events.

In this study, TLV is employed as the retrieval unit in the RAG pipeline. Depending on how log representations within a trace are constructed, TLV supports both single-chunk and multi-chunk configurations. We quantitatively compare these configurations to analyze how different trace-level chunking strategies affect retrieval performance in system log analysis.

### 2.7. Chunk as Relational Data (CARD)

In RAG-based system log analysis, the definition of a retrieval chunk is a critical design factor that directly affects retrieval performance. Conventional approaches typically define chunks as simple text segments, embedding log messages by sequentially concatenating textual content. However, system logs are not merely collections of independent text events; rather, they represent execution records in which multiple entities and invocation relationships are accumulated over time. As a result, purely text-based chunking strategies struggle to adequately capture execution flows and relational information inherent in system logs.

To overcome these limitations, this study introduces Chunk as Relational Data (CARD) as a design principle for defining retrieval units. CARD treats a chunk not as a fragmented piece of text but as a relational execution unit reconstructed from the perspective of specific entities. Each CARD-based chunk encapsulates invocation relationships and inter-entity interactions observed within an execution flow, forming a coherent logical object that preserves contextual dependencies.

CARD serves as the foundational unit for constructing TLV representations. Under this principle, differences between single-chunk and multi-chunk strategies can be systematically explained by the scope and granularity at which CARD is applied within a trace. This design enables flexible control over how execution flows and entity relationships are represented and retrieved in RAG-based system log analysis.

The end-to-end procedure for constructing Single-Chunk and Multi-CARD representations and building the retrieval index is summarized in Algorithm 1.
**Algorithm 1** LogVector Index Construction (Single-Chunk / Multi-CARD)Require: CSV file D, mode ∈ {Single, Multi}, flags NormalizeSQL, MaskPII, embedding model M, UseTFIDF (optional), max rows Nmax (optional) Ensure: FAISS index I, chunk store (cards, ids, meta) 1: df ← ReadCSV(D)2: df ← df[0 : min(|df|, Nmax)]    ▷ Optional truncation3: if mode = Single then4:   C ← DetectSchemaColumns(df)    ▷ Hint-based auto-detection5:   cards ← [ ], ids ← [ ]6:   for all row r in df with index i do7:      s ← ∅8:      for all column c ∈ C do9:         v ← r[c]10:        v ← NormalizeGeneric(v, NormalizeSQL, MaskPII, c)11:        if v ≠ ϕ then12:            s ← s ∥ "c: v"    ▷ Key-Value formatting13:         end if14:      end for15:      cards.append(s)16:      ids.append(RowID(r, i))17:   end for18:else if mode = Multi then19:   cards ← [ ], ids ← [ ]20:   G ← {table, Call_Func}    ▷ Grouping entities (Anchor)21:   for all gcol ∈ G do22:      df’ ← DropNA(df, gcol)23:      for all group H in GroupBy(df’, gcol) do24:         gval ← FirstValue(H, gcol)25:         T ← Unique(H[table])    ▷ Related tables (Target)26:         F ← Unique(H[Call_Func])    ▷ Related functions (Target)27:         P ← Unique(H[Call_File])    ▷ Contextual metadata28:         T, F, P ← NormalizeSets(T, F, P, MaskPII)29:         if gcol = table then30:            s ← "ENTITY_TYPE:TABLE ENTITY_VALUE:" ∥ gval31:            s ← s ∥ " ACCESSED_BY_FUNCTIONS:" ∥ Join(F)32:            s ← s ∥ " ACCESSED_BY_FILES:" ∥ Join(P)33:         else if gcol = Call_Func then34:            s ← "ENTITY_TYPE:FUNCTION ENTITY_VALUE:" ∥ gval35:            s ← s ∥ " ACCESSES_TABLES:" ∥ Join(T)36:            s ← s ∥ " CALLED_FROM_FILES:" ∥ Join(P)37:         end if38:         cards.append(s)39:         ids.append(gcol : gval)40:      end for41:   end for42:end if43:(X, meta) ← BuildEmbeddings(cards, M, UseTFIDF)    ▷ L2-normalized vectors44:I ← FAISS_IndexFlatIP(dim(X))    ▷ Inner Product for cosine similarity45:I.add(X)46:Save(I, cards, ids, meta)
47:return I, (cards, ids, meta)

### 2.8. Single-Chunk and Multi-Chunk Retrieval Design

When Trace-LogVector (TLV) is used as the retrieval unit in a RAG-based system log analysis framework, the manner in which a trace is transformed into textual representations for embedding becomes a critical design factor. In particular, system log analysis involves heterogeneous query types, including queries targeting entire execution flows as well as queries focused on specific entities. Therefore, it is necessary to consider how different trace representation strategies influence retrieval characteristics.

**Figure 2:**
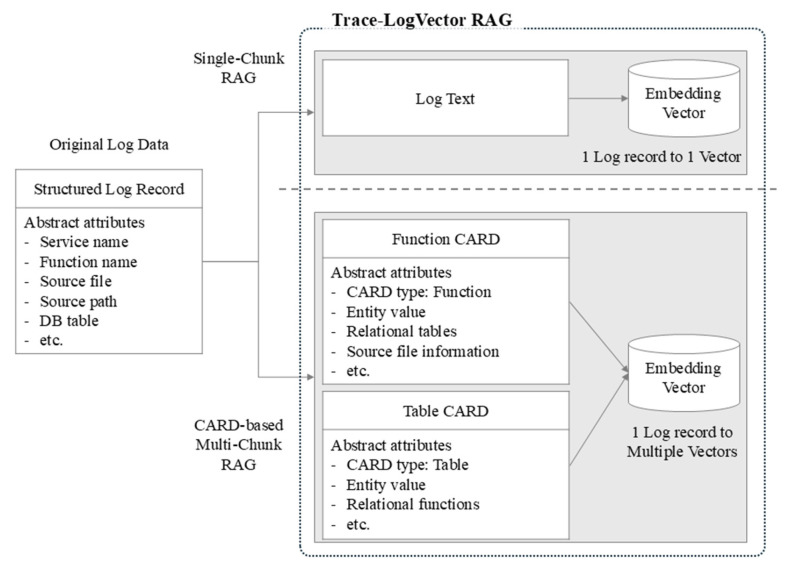
Conceptual comparison of single-chunk and CARD-based multi-chunk representations.

As illustrated in Figure 2, this study defines two distinct trace representation strategies: a single-chunk approach and a CARD-based multi-chunk approach. The single-chunk approach embeds all log events belonging to a trace as a single textual unit, whereas the multi-chunk approach reconstructs trace information into structured CARD representations based on entity perspectives and embeds each CARD independently.

#### 2.8.1. Single-Chunk Representation

In the single-chunk approach, all log events associated with a trace are concatenated in execution order and provided as input to the embedding model, resulting in a single vector representation per trace. The retrieval unit corresponds to the entire execution flow of a trace rather than individual log lines. No explicit decomposition based on entity perspectives is applied, and the embedding model internally normalizes the input text into a fixed-dimensional vector representation.

This approach offers low preprocessing overhead and implementation simplicity. By aggregating the full execution context into a single vector, it is suitable for queries that target overall processing flows of individual requests, such as end-to-end transaction paths or execution sequences surrounding error occurrences. However, as trace length increases, entity-specific signals—such as particular function calls or database accesses—may become less distinguishable within the aggregated representation, limiting expressiveness for queries that require fine-grained relational analysis.

#### 2.8.2. Multi-Chunk Representation Based on CARD

In the multi-chunk approach, execution events within a trace are analyzed from entity-centric perspectives and reconstructed into CARD-based representations prior to embedding. Each CARD is defined as a relational execution unit expressed in a structured, key–value-oriented textual format. CARDs are generated based on entities extracted from service call analysis results, and each CARD is treated as an independent embedding input.

In this study, two representative CARD types are constructed: Table Cards and Function Cards (see Appendix A for concrete examples). A Table Card designates a specific database table as the primary entity and represents related functions, files, and invocation paths as relational attributes. Conversely, a Function Card treats a specific function as the primary entity and describes associated tables and invocation contexts. In both cases, inter-entity relationships are explicitly preserved within the textual representation. Consequently, a single trace can be represented as a collection of multiple CARD vectors, reflecting different entity perspectives of the same execution flow. This process extends the representation space by decomposing a trace into relational units rather than performing simple text segmentation.

#### 2.8.3. Experimental Design Considerations

To isolate the impact of trace representation strategies on retrieval behavior, TLV representations are constructed using identical trace datasets and query sets for both single-chunk and multi-chunk configurations. All experimental factors other than chunking strategy—including the embedding model, vector dimensionality, similarity function, and retrieval algorithm—are held constant. The independent variable is limited to the trace representation strategy, enabling controlled analysis of how single-chunk and CARD-based multi-chunk designs influence retrieval behavior.

Retrieval performance is evaluated using Hit@K and Mean Reciprocal Rank (MRR) metrics, computed as average values across all queries.

### 2.9. RAG Pipeline Architecture

**Figure 3:**
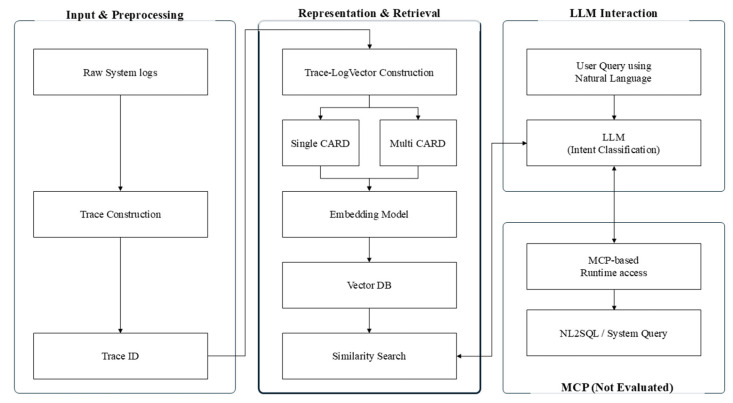
Overall architecture of the proposed conversational system log analysis pipeline.

The RAG pipeline employed in this study is designed with a clear separation between the retrieval stage and the generation stage, as illustrated in Figure 3. This design choice enables quantitative analysis of the impact of TLV chunking strategies on retrieval performance while isolating effects introduced by the generation process. The overall architecture consists of a retrieval layer based on relational log representations and an execution layer designed to support real-time system interactions. In this study, experimental evaluation is restricted to the retrieval layer, and neither the generation stage nor the execution layer is included in the performance assessment.

#### 2.9.1. RAG Pipeline Overview

The pipeline comprises four main stages: (1) log preprocessing and trace construction, (2) TLV generation, (3) vector-based retrieval, and (4) result output. First, traces are constructed from raw logs using request identifiers or invocation flow information. Each trace is then transformed into TLV representations using either a single-chunk or a multi-chunk strategy and embedded into vector representations via an embedding model. The resulting vectors are stored in a vector database.

Upon receiving a query, the same embedding model is used to encode the query into a vector representation. Top-K TLVs are retrieved using cosine similarity-based nearest neighbor search. In this study, the retrieval output itself is treated as the final result, and the linguistic quality of generated responses is not evaluated.

#### 2.9.2. MCP-Based Execution Layer (Not Evaluated)

The proposed overall architecture includes an execution layer designed to support real-time system interactions based on retrieval results. This layer leverages the MCP to enable secure interactions with external system resources, such as database queries, system state inspection, and additional log collection. However, as the experimental evaluation in this study focuses exclusively on retrieval performance, the accuracy and performance of the MCP-based execution layer are not evaluated. MCP is included as an architectural component to illustrate the extensibility of the proposed conversational system analysis framework.

#### 2.9.3. Experimental Data and Query Design

Experiments are conducted using trace datasets extracted from real-world system logs. Each dataset contains entity information relevant to system analysis, including service identifiers, function invocations, and database access records. Queries are designed to explore error causes, invocation paths, or entity relationships. For each query, a ground-truth trace or a ground-truth entity set is predefined to enable quantitative evaluation of retrieval correctness.

#### 2.9.4. Experimental Configuration and Variable Control

To compare single-chunk and multi-chunk strategies, identical trace datasets, embedding models, and vector retrieval configurations are used across all experimental conditions. The chunking strategy is treated as the sole independent variable, while all other factors are strictly controlled. Retrieval performance is evaluated using Hit@K and Mean Reciprocal Rank (MRR) metrics, with values computed as averages across all queries.

### 2.10. Experimental Setup

The experiments in this study are designed to isolate the impact of chunking strategies on retrieval performance within a TLV-based RAG framework. Rather than optimizing the embedding model itself, we analyze retrieval performance variations caused solely by differences in log representation units. Therefore, all experiments use the same data source, the same embedding model, and identical vector retrieval configurations, treating the representation strategy (chunking) as the primary independent variable for the main single vs. multi comparison.

We employ a Transformer-based sentence embedding model for vectorization without any additional training or fine-tuning beyond the pretrained weights. Vector retrieval is performed via cosine-similarity nearest-neighbor search, and Top-K results are returned for each query.

The end-to-end procedure is summarized in Algorithm 1 (index construction), Algorithm 2 (query encoding), and Algorithm 3 (retrieval evaluation with Hit@K and MRR@K). The corresponding pseudocode is provided below (see Appendix C for further reproducibility details).
**Algorithm 2** Query Encoding for RetrievalRequire: query q, embedding meta meta, model name M, flags NormalizeSQL, MaskPII Ensure: query vector z ∈ R^d^ 1:  q' ← NormalizeGeneric(q, NormalizeSQL, MaskPII) 2:  if meta.type = st then 3:      z ← SentenceTransformerEncode(M, q') 4:      z ← L2Normalize(z) 5:  else if meta.type = tfidf then 6:      X_c_ ← meta.vec_char.transform(q') 7:      X_w_ ← meta.vec_word.transform(q') 8:      X ← [X_c_; X_w_]    ▷ concatenate sparse features 9:      X ← L2Normalize(X) 10:    z ← ToDense(X) 11: end if 12: return z
**Algorithm 3** RAG Retrieval Evaluation (Hit@K, MRR@K)Require: FAISS index I, chunk ids ids, embedding meta meta, evaluation set  Q = ${\left\{(q_{i},\;E_{i})\right\}}_{i=1}^{n}$ where E_i_ is expected_ids, top-K Ensure: Hit@K, MRR@K 
1:  HITS ← 0, RR ← [ ]
2:  for i = 1 to n do
3:      z_i_ ← EncodeQuery(q_i_, meta)    ▷ Alg. 2
4:      (_, I_i_) ← I.search(z_i_, K)
5:      R_i_ ← [ids[j] : j ∈ I_i_]    ▷ retrieved ids
6:      if R_i_ ∩ E_i_ ≠ ∅ then
7:          HITS ← HITS + 1
8:      end if
9:      r ← 0
10:    for t = 1 to K do
11:          if R_i_[t] ∈ E_i_ then
12:              r ← t; break
13:          end if
14:      end for
15:      if r > 0 then
16:          RR.append(1/r)
17:      else
18:          RR.append(0)
19:      end if
20: end for
21: Hit@K ← HITS/n
22: MRR@K ← mean(RR)
23: return Hit@K, MRR@K

For reproducibility, the source code and experimental datasets are publicly released via GitHub to support further analysis of the proposed Trace-LogVector-based RAG framework [19].

#### 2.10.1. Dataset Construction

The dataset used in our experiments is obtained from a widely used open-source web-application backend (WordPress, version 6.9.3), providing a publicly reproducible representative backend model. We use this dataset as a controlled proxy to reproduce execution-log characteristics that commonly arise when sensor telemetry is integrated into application services and persistent storage, without claiming it is identical to device-side IoT logs. Table 1 summarizes the actual schema used in our implementation. Each row corresponds to a discrete execution record, capturing (i) the source-code context (Call_File), (ii) the invoked function signature (Call_Func), (iii) the accessed data-store entity (table), and (iv) the preprocessed textual payload (card_text) used for vector embedding.

Although the dataset originates from a web-application backend, the representation is domain-agnostic because it relies on generic execution entities (function, data-store, and source module) and their relations—fields that are commonly present in the operational-log layer of sensor-driven services. This schema provides a structured foundation for constructing entity-centric CARD representations, as summarized in Algorithm 1.

The experimental dataset is derived from service call analysis results generated by a domain boundary analysis system proposed in prior work [18], which extracts service invocation relationships, function call information, and database access patterns from backend execution logs. In this study, the resulting relational call-log records are used as the primary data source for evaluation.

The dataset consists of 7572 relational execution records, each representing a structured association between an invoked function and an accessed database table, along with corresponding source file information. Each log record includes structured fields describing invocation relationships, such as accessed database tables, invoked functions, and source files. During preprocessing, unnecessary textual elements are removed, and numeric values and identifiers are masked to improve the stability and robustness of embedding inputs.

To enable a controlled comparison, two different indexing units are constructed from the same data source based solely on the chunking strategy. In the single-chunk configuration, each relational execution record is represented as a single textual chunk and treated as an independent retrieval unit. In contrast, the multi-chunk configuration reconstructs execution events into structured relational indices by generating Table Cards and Function Cards derived from the same service call analysis results. This process does not simply split logs into smaller text fragments; rather, it reconstructs identical execution events from different entity-centric perspectives.

From a dataset composition perspective, the single-chunk strategy embeds all collected relational execution records, resulting in 7572 retrieval units. The multi-chunk strategy, however, selectively reconstructs entity-centric CARD representations only for execution flows that explicitly contain function–table relationships, in accordance with the TLV definition. This design choice intentionally excludes redundant patterns and unstructured log messages, enabling the construction of a gold-standard relational dataset centered on unique execution flows with structural completeness. Given that complex execution flows and transactional behaviors in IoT cloud architectures primarily occur on server-side components, web application server (WAS) logs are used as representative trace-level datasets in this study.

#### 2.10.2. Query and Ground-Truth Definition

A set of five analysis-oriented queries is constructed to reflect scenarios frequently encountered in real-world system operation tasks. Rather than simple keyword-based searches, the queries are designed to require understanding of execution flows and entity relationships, such as tracing the root cause of specific errors, identifying service invocation paths, and analyzing function–table associations.

For each query, the ground truth is defined not as a single sentence but as a corresponding execution trace or a set of related entities directly relevant to the analysis objective (see Appendix B for concrete examples). Ground-truth labels are manually verified based on service call analysis results and predefined execution flow relationships. This design allows retrieval correctness to be evaluated using both binary relevance judgments (i.e., whether the correct trace or entity set is included in the retrieved candidates) and rank-based metrics.

Although the number of evaluation queries is limited, the retrieval corpus consists of 7572 relational execution records, enabling each query to be evaluated against a non-trivial and realistic search space. The query–ground-truth design prioritizes accurate retrieval of essential execution flows and relational contexts over the linguistic quality of generated responses, thereby aligning the evaluation methodology with the analytical requirements of system log analysis.

#### 2.10.3. Embedding and Vector Retrieval Configuration

Both queries and log representations are embedded using the same encoder. Non-natural log records are rendered into semi-structured text (Single-Chunk text or CARD templates) so that a transformer-based sentence embedding model can project natural-language queries and structured log text into a shared semantic space. This design reduces modality mismatch between natural-language queries and execution logs and supports cross-modal semantic matching between unstructured human queries and structured log templates, moving beyond exact keyword overlap.

We use a pretrained sentence embedding model from the SentenceTransformers framework(version 5.2.3, https://sbert.net), specifically a MiniLM-based Transformer (L6). No additional training or fine-tuning is applied, so the reported results reflect differences due to representation design and retrieval settings rather than model adaptation.

In addition to the transformer-based retriever, we include a TF-IDF-based lexical retriever as a traditional non-neural baseline. This lexical baseline uses the same corpus and query set and is evaluated under the same representation settings (Single-Chunk vs. Multi-CARD), enabling a controlled comparison between frequency-based matching and semantic matching.

All vectors are indexed using FAISS (Facebook AI Similarity Search, version 1.13.2), and nearest-neighbor retrieval is conducted using cosine similarity. For each query, the Top-5 results (K = 5) are returned. Retrieval performance is evaluated using Hit@5 and Mean Reciprocal Rank at 5 (MRR@5). Overall, this configuration controls for data source and evaluation protocol, while allowing us to analyze the effects of representation design and retriever choice in a systematic manner.

#### 2.10.4. Controlled Comparison Design

To ensure a fair comparison between single-chunk and multi-chunk strategies, both experimental conditions use identical log datasets and the same query sets. The chunking strategy used to construct trace representations is treated as the primary independent variable, while all other factors—including the embedding model, vector dimensionality, similarity metric, and retrieval algorithm—are strictly controlled. This design ensures that performance differences in the main comparison are attributable to TLV chunk construction.

## 3. Results

This section analyzes retrieval performance differences between single-chunk and multi-chunk strategies in a TLV-based RAG framework using Hit@K and MRR@K (K = 5). All experimental results are obtained under controlled conditions using the same log dataset, query set, embedding configuration, and retrieval settings, while treating the representation strategy (chunking) as the primary independent variable.

To strengthen the comparison, we additionally report (i) a standard sliding-window chunking baseline with a chunk-size sensitivity analysis using a contextual-purity metric (Noise), and (ii) a 2 × 2 ablation study across Representation (Single vs. Multi-CARD) and Retriever (TF-IDF vs. Transformer), as summarized in Section 3.4.

### 3.1. Hit@K Results

Table 2 presents the Hit@5 results for the single-chunk and multi-chunk strategies. The single-chunk TLV achieved a Hit@5 score of 0.6000, indicating that the correct trace was included within the top five retrieved results for only three out of five queries. In contrast, the multi-chunk TLV achieved a Hit@5 score of 1.000, meaning that the correct chunk was included within the top five results for all queries.

### 3.2. MRR Results

Table 3 presents the MRR@5 results obtained under the same experimental conditions. The single-chunk TLV achieved an MRR@5 score of 0.2167, indicating that the correct trace was ranked relatively low, at approximately the 4.6th position on average. In contrast, the multi-chunk TLV achieved an MRR@5 score of 0.900, meaning that the correct chunk was typically ranked within the top one or two positions.

Unlike Hit@K, MRR emphasizes ranking quality by measuring how early the correct result appears. The substantial improvement in MRR achieved by the multi-chunk strategy suggests that relationally decomposed CARD representations enable more precise semantic alignment between queries and relevant execution information. By explicitly encoding localized entity relationships, the multi-chunk strategy encourages query-relevant information to be positioned closer in the vector space.

### 3.3. Summary of Results

Taken together, the Hit@5 and MRR@5 results confirm that chunking strategy is a critical design factor in TLV-based retrieval. The multi-chunk strategy consistently outperforms the single-chunk approach across both metrics, indicating not only improved recall but also enhanced ranking precision.

These findings suggest that representing system logs as relational contextual units—rather than as monolithic text sequences—can substantially improve both recall and precision in RAG-based retrieval. More broadly, the results demonstrate that the definition and scope of retrieval units play a decisive role in retrieval effectiveness, particularly in domains where queries target relationships and execution flows rather than isolated textual facts. This observation aligns conceptually with prior work in information retrieval emphasizing the impact of representation granularity on retrieval performance [20,21].

### 3.4. Additional Baselines and Sensitivity Analysis

Table 4 presents the sensitivity analysis results for the naive sliding-window chunking baseline across different chunk sizes. As the chunk size increases from 100 to 1000 characters, the Noise metric rises from 2.00 to 7.16, indicating that larger temporal segments increasingly mix unrelated execution rows into a single retrieved evidence block. Although Hit@5 remains saturated at 1.0 due to the large ground-truth sets (avg. 895 rows/query; min = 128, max = 1391), the ranking quality varies across configurations, as reflected by MRR@5 (0.7000 at 100, 0.5667 at 500, and 0.6667 at 1000). These results quantitatively demonstrate the context-pollution limitation of standard chunking in high-concurrency environments.

Table 5 reports a 2 × 2 ablation study across Representation (Single-Chunk vs. Multi-CARD) and Retriever (TF-IDF vs. Transformer) to separate the effect of representation structure from retriever choice. Under TF-IDF, the Single-Chunk representation achieves Hit@5 = 0.2000 and MRR@5 = 0.1000, whereas the Multi-CARD representation achieves Hit@5 = 1.0000 and MRR@5 = 0.7167. Under the Transformer-based retriever (TLV), the Single-Chunk representation achieves Hit@5 = 0.6000 and MRR@5 = 0.2167, while Multi-CARD achieves Hit@5 = 1.0000 and MRR@5 = 0.9000. Notably, the improvement of Multi-CARD under TF-IDF indicates that the primary gain comes from relational restructuring rather than from using a deep embedding model alone.

## 4. Discussion

### 4.1. Interpretation of Chunking Strategy Effects

Our sliding-window sensitivity analysis in Table 3 shows that the Noise metric increases monotonically with chunk size, rising from 2.00 (100 chars) to 7.16 (1000 chars). This indicates that larger temporal segments increasingly interleave multiple unrelated execution rows into a single retrieved evidence block, reducing contextual purity. These observations motivate CARD-based relational chunking, which isolates logically related interactions into more coherent evidence units for LLM-assisted analysis.

The experimental results demonstrate that representation strategy substantially influences retrieval performance in TLV-based RAG systems. This finding suggests that when log data are treated as execution-centric analytical units containing both flow and relational signals, the scope of those units directly affects semantic matching during retrieval. This observation is consistent with recent studies emphasizing that retrieval-stage design plays a dominant role in overall RAG effectiveness, often preceding generation quality in importance [13].

The single-chunk strategy encodes an entire trace as a single vector representation. This approach can be suitable for queries that require broad trace-level coverage. However, as trace length increases, heterogeneous events and entities are aggregated into one representation, which can dilute entity-specific signals and reduce discriminative power for relation-centric queries. As a result, relevant traces may still be retrieved but ranked lower.

In contrast, the multi-chunk strategy decomposes traces into smaller, entity-centric units. By embedding each CARD within a constrained relational scope, entity- and relation-specific signals are preserved more explicitly, improving sensitivity to targeted queries about function–data-store interactions. These findings support defining retrieval chunks as relational analytical units rather than as simple text partitions.

### 4.2. Relationship Between Query Characteristics and TLV

Unlike conventional document retrieval, system analysis queries are typically analytical in nature, focusing on causal reasoning, execution path tracing, and relationship exploration. These characteristics necessitate careful consideration of retrieval unit design.

TLV addresses this requirement by treating traces as the minimal analytical unit, reflecting the domain reality that system analysis often involves identifying “correct execution units” rather than isolated textual answers. The experimental results indicate that TLV-based representations can effectively support RAG retrieval, while also revealing that different chunking strategies may be better suited to different query types.

These observations suggest that retrieval units should not be statically defined but adaptively selected based on query characteristics. TLV provides a flexible representation framework capable of accommodating both holistic and entity-centric retrieval needs. This perspective aligns with recent RAG evaluation studies that emphasize prioritizing retrieval correctness over surface-level linguistic similarity [14].

### 4.3. Limitations and Future Work

This study focuses on analyzing the impact of chunking strategies on retrieval performance in TLV-based RAG systems, with evaluation restricted to retrieval-stage metrics such as Hit@K and Mean Reciprocal Rank (MRR). Accordingly, the linguistic quality of generated responses and the operational efficiency of end-to-end system deployments are beyond the scope of the current evaluation.

In addition, the experimental evaluation is conducted using a limited set of analysis-oriented queries under a controlled setting. This design choice was made to isolate the effect of log representation and chunking strategy while holding all other variables constant, rather than to provide a large-scale benchmark of query distributions. Each query is evaluated against a non-trivial retrieval corpus consisting of 7572 relational execution records, ensuring that retrieval performance is assessed over a realistic and sufficiently large search space.

A limitation is that our evaluation data originates from a web-application backend and thus does not directly cover all device-side IoT logging stacks or proprietary protocols. Nevertheless, TLV/CARD are designed to operate on extracted relational entities (e.g., function–data-store interactions) rather than domain-specific semantics. Future work will validate the approach on additional datasets from IoT gateways and cloud-native microservices to further strengthen cross-domain generality.

The experiments are also based on a specific system architecture and logging format, which may limit direct generalization to other environments. Future work will extend the evaluation to larger and more diverse query sets, as well as to different system architectures and logging formats. In addition, adaptive chunk selection mechanisms based on query intent and tighter integration between TLV-based retrieval and real-time execution layers may further enhance the applicability of the proposed framework. Such extensions naturally connect to emerging research on tool-augmented LLMs and retrieval–execution hybrid architectures [22].

## 5. Conclusions

This study redefines system logs generated in IoT-based cloud environments—where numerous sensors and distributed services interact—as relational analytical units at the execution-flow (trace) level rather than as collections of isolated events. Based on this perspective, we quantitatively analyzed the impact of chunking strategies on retrieval performance in a Trace-LogVector (TLV)-based Retrieval-Augmented Generation (RAG) framework. Specifically, retrieval performance was evaluated by comparing single-chunk and multi-chunk strategies using Hit@K and Mean Reciprocal Rank (MRR) metrics. The results empirically demonstrate that preserving execution flows and entity relationships as retrievable analytical units enables system log analysis in IoT cloud environments to be effectively reframed as a RAG-based conversational information retrieval problem.

Experimental results confirm that chunking strategy is a critical design factor in TLV-based retrieval. While the single-chunk strategy is effective in maintaining the global context of an entire execution flow within a single vector representation, the multi-chunk strategy provides finer-grained representations of localized execution segments and entity-specific interactions. Consequently, the multi-chunk approach achieves higher retrieval alignment for entity and relationship-centric queries. These findings suggest that the choice of log representation granularity and chunking strategy should be guided by the characteristics of system analysis queries, and they highlight the importance of constructing logs as relational contextual units rather than as plain textual sequences.

The contributions of this study can be summarized as follows.

1. First, we introduce the concept of Trace-LogVector (TLV), which explicitly incorporates execution flows and entity relationships embedded in system logs and defines trace-level representations as the minimal analytical unit for RAG-based retrieval.
2. Second, we propose the design principle of Chunk as Relational Data (CARD), which treats chunks as relational analytical units rather than simple text partitions, and we quantitatively compare single-chunk and multi-chunk strategies within this framework.
3. Third, by focusing evaluation on retrieval accuracy and ranking quality rather than on the linguistic quality of generated responses, we present a retrieval-centric performance evaluation perspective that is well aligned with the requirements of system log analysis.

This study focuses primarily on retrieval-stage performance and does not evaluate the quality of generated responses or the operational efficiency of deployment in production environments. In addition, the experiments were conducted using a specific log dataset and query set, which may limit generalizability across diverse system architectures and logging formats. Future work may explore adaptive chunk selection strategies based on query intent, as well as the integration of TLV-based retrieval with interactive real-time execution layers—such as those enabled by Model Context Protocol (MCP)—to further extend the practicality of conversational system log analysis frameworks.

## Figures and Tables

**Table 1:** Schema of the extracted backend execution-log dataset used in our experiments.

Column	Data Type	Description
id	Integer	Unique ID for each backend execution record (row-level unit).
Call_File	String	Source code file path where the execution logic is triggered.
Call_Func	String	Invoked function signature executing backend logic; used as a primary grouping anchor in Algorithm 1.
table	String	Accessed data-store entity (database table) used for state persistence/retrieval.
card_text	String	Preprocessed relational text payload directly used for vector embedding and retrieval.

**Table 2:** Hit@5 comparison between single-chunk and multi-chunk Trace-LogVector.

TLV Type	Hit@5	Interpretation
Single-chunk TLV	0.6000	Correct trace included in Top-5 for 3 out of 5 queries
Multi-chunk TLV	1.0000	Correct trace included in Top-5 for all queries

**Table 3:** MRR@5 comparison between single-chunk and multi-chunk Trace-LogVector.

TLV Type	MRR@5	Interpretation
Single-chunk TLV	0.2167	Correct trace ranked at 4.6 on average
Multi-chunk TLV	0.9000	Correct trace ranked within top 1–2 on average

**Table 4:** Sensitivity analysis of the naive sliding-window baseline.

Chunk Size	Overlap	Hit@5	MRR@5	Noise (Rows/Chunk)
100	20	1.0	0.7000	2.00
300	50	1.0	0.6667	3.08
500	50	1.0	0.5667	4.24
1000	100	1.0	0.6667	7.16

**Table 5:** 2 × 2 ablation: Representation × Retriever.

Retriever	Single-Chunk	Multi-CARD
TF-IDF	Hit@5 = 0.2000; MRR@5 = 0.1000	Hit@5 = 1.0000; MRR@5 = 0.7167
Transformer (TLV)	Hit@5 = 0.6000; MRR@5 = 0.2167	Hit@5 = 1.0000; MRR@5 = 0.9000

## Data Availability

The data and source code presented in this study are available publicly on GitHub at https://github.com/sun-iron/trace-logvector (accessed on 10 March 2026).

## Appendix A. Example of CARD-Based Relational Representations

This appendix provides concrete examples of the Chunk as Relational Data (CARD) representations used in the multi-chunk TLV configuration. The purpose of this appendix is to illustrate how execution-flow-level log records are transformed into entity-centric relational retrieval units, rather than simple text fragments.

### Appendix A.1. Table Card Example

Entity: wp_usermeta (Database Table)

Relational Context:

- Associated Functions:

update_meta_cache(), get_user_meta(), delete_user_meta()

- Source Files:

wp-includes/meta.php

- Invocation Characteristics:

Invoked during user authentication and profile update workflows

- Execution Scope:

Server-side transactional operations related to user metadata access

**Description:**

This Table Card represents the relational execution context of the wp_usermeta table. Instead of listing isolated log lines, the card encapsulates all functions and invocation paths associated with this table within a given execution flow. As a result, queries targeting table-centric relationships can retrieve this card as a coherent analytical unit.

### Appendix A.2. Function Card Example

Entity: WP_Post::get_instance() (Function)

Relational Context:

- Accessed Tables:

wp_posts

- Source Files:

wp-includes/class-wp-post.php

- Invocation Path:

Post retrieval and rendering pipeline

- Execution Scope:

Read-only access to post metadata during request handling

Description:

This Function Card captures the relational context of the function WP_Post::get_instance(). The card aggregates information about accessed tables, source files, and invocation paths, enabling function-centric queries to retrieve execution-relevant information as a single relational unit.

## Appendix B. Example of Query and Ground-Truth Definition

This appendix illustrates how analysis-oriented queries are mapped to ground-truth retrieval targets in the evaluation process.

### Appendix B.1. Query-to-Ground-Truth Mapping Example

Query: Which functions access the wp_usermeta table?

Ground Truth**:** table:wp_usermeta

Rationale:

The evaluation targets retrieval of the Table Card representing wp_usermeta, which encapsulates all associated functions and execution contexts. Retrieval correctness is therefore defined by whether the relational execution unit (CARD) corresponding to the table is included in the retrieved results, rather than by literal enumeration of function names.

### Appendix B.2. Function-Centric Query Example

Query: Which table(s) does the function WP_Post::get_instance() access?

Ground Truth: Call_Func:WP_Post::get_instance()

Rationale:

Although the query mentions accessed tables, the correct retrieval target is the Function Card itself, as it encapsulates the relational context (including table access) of the function. This design reflects the retrieval-centric evaluation objective of the study.

## Appendix C. Pseudocode for Reproducibility

Time complexity note: assuming hash-based grouping, index construction scans

*N* records and performs grouping in expected *O*(*N*) time; Single-Chunk construction additionally concatenates selected columns per row.

Algorithms 1–3 summarize the end-to-end procedure for constructing Single-Chunk and Multi-CARD representations, query encoding, and retrieval evaluation.

**Appendix Summary**

These examples demonstrate that CARD-based TLV representations are relational, execution-aware retrieval units, rather than flat text chunks. By defining ground truth at the level of relational execution units, the evaluation framework aligns retrieval correctness with the analytical requirements of system log analysis.
